# The Accuracy of the PREP2 Prediction Tool for Upper Limb Outcomes After Stroke as Part of Routine Clinical Care

**DOI:** 10.1177/15459683251412283

**Published:** 2026-01-23

**Authors:** Harry Jordan, Olivia Norrie, Cathy M. Stinear

**Affiliations:** 1 Clinical Neuroscience Laboratory, Department of Medicine, The University of Auckland, Auckland, New Zealand; 2 Centre for Brain Research, University of Auckland, Auckland, New Zealand; 3 Allied Health, Te Whatu Ora Health New Zealand Te Toka Tumai Auckland, Auckland, New Zealand

**Keywords:** stroke, upper limb, biomarkers, prediction, transcranial magnetic stimulation, MEP status

## Abstract

**Background:**

The Predict REcovery Potential-2 (PREP2) prediction tool uses clinical assessments and transcranial magnetic stimulation (TMS) within 1 week post-stroke to predict individuals’ upper limb functional outcome at 3 months (3M) post-stroke. PREP2 was successfully implemented in clinical care at Auckland City Hospital, New Zealand in 2017.

**Objective:**

The primary aim was to evaluate the accuracy of PREP2 predictions made by clinicians during routine clinical care, with a threshold of 70% accuracy for validation. A secondary aim was to identify new baseline predictors that could increase PREP2 accuracy.

**Methods:**

Eighty-three patients who received PREP2 predictions were recruited within 1 week of stroke and had their upper limb outcome assessed at 3M post-stroke with the Action Research Arm Test. Cognition and sensation were evaluated within 1 week of stroke.

**Results:**

Overall accuracy of the PREP2 prediction tool in clinical practice was 66% (95% confidence interval, 55%-76%). Accuracy was highest for the Excellent (80%) and Poor (100%) categories and lowest for the Good category (36%). Prediction accuracy for Good outcomes was 67% for patients who did not require TMS and 27% for patients who did. Finger extension differentiated participants predicted to have a Good outcome using TMS who did and did not have a favorable upper limb outcome.

**Conclusions:**

Excellent and Poor predictions are highly accurate when used in clinical practice, however the full PREP2 tool is not yet validated in clinical practice. Future studies with larger samples could investigate additional measures to enhance accuracy of the Good prediction category.

Clinical trial registration number ACTRN12619000225112, https://anzctr.org.au/Trial/Registration/TrialReview.aspx?ACTRN=12619000225112.

## Introduction

**Figure 1.**
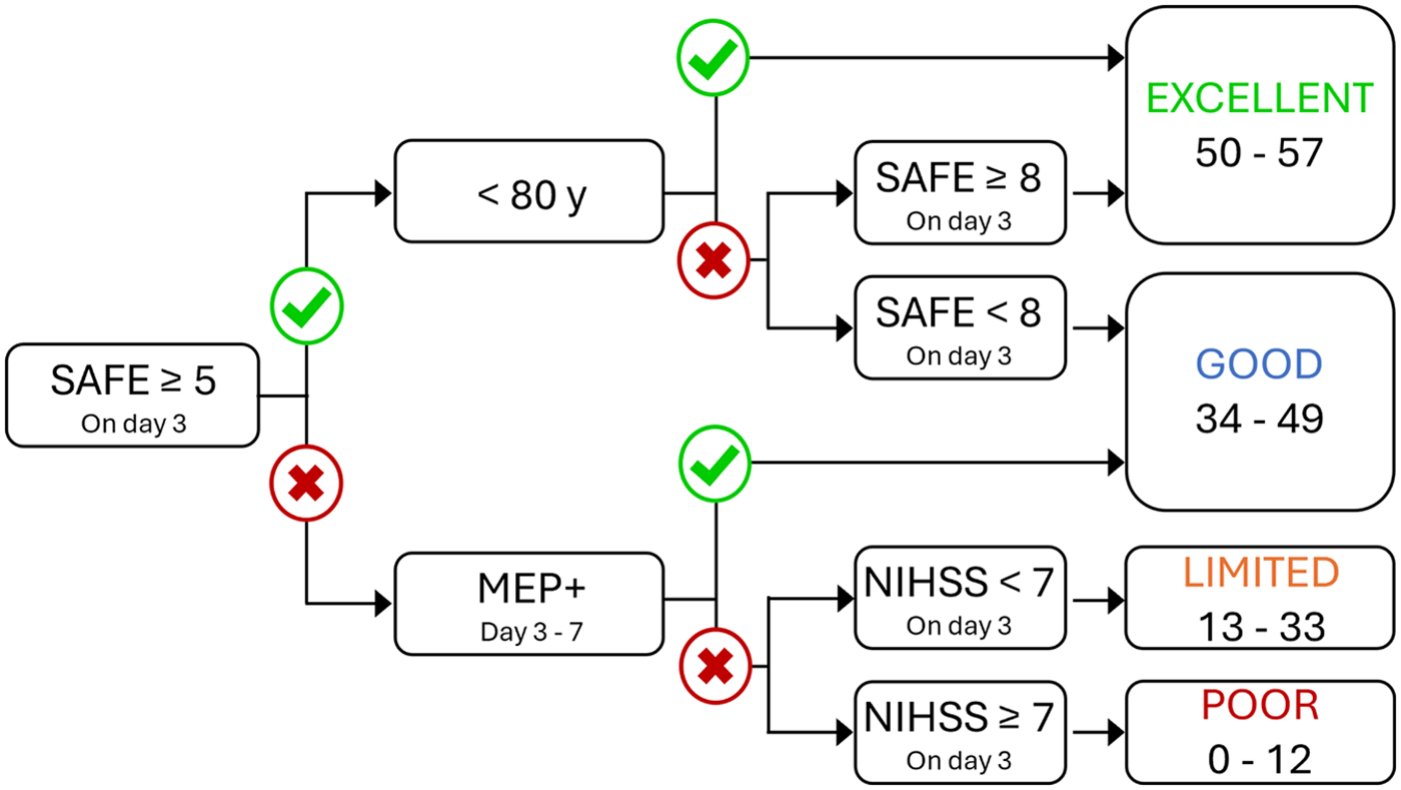
The PREP2 tool for predicting upper limb functional outcome at 3 months post-stroke. There are 4 possible predictions depending on the person’s SAFE score, age, MEP status, and NIHSS score. A SAFE score on day 3 post-stroke is obtained for all patients with upper limb weakness and they proceed through the tool until a prediction is reached. The outcome boxes on the right provide Action Research Arm Test score ranges for each outcome category. Abbreviations: PREP2, predict REcovery potential-2; SAFE, Shoulder Abduction Finger Extension, MEP: motor evoked potential; NIHSS, National Institutes of Health Stroke Scale.

Stroke is a leading cause of long-term adult disability worldwide and approximately 50% of people experience upper limb impairment immediately after a stroke.^1,2^ Predict REcovery Potential-2 (PREP2) is a prediction tool that combines clinical and neurophysiological assessments within the first week after stroke to predict an individual’s upper limb functional outcome at 3 months (3M) post-stroke (Figure 1).^3^ PREP2 predicts 1 of 4 upper limb outcomes: Excellent, Good, Limited, or Poor.

The first step for PREP2 is to establish a patient’s Shoulder Abduction Finger Extension (SAFE) score. The SAFE score is obtained by assessing paretic shoulder abduction (SA) and finger extension (FE) strength using the Medical Research Council (MRC) strength grades. The MRC grades for each movement are scored out of 5 and then summed to obtain a SAFE score out of 10. Patients with a SAFE score of at least 5 before or on day 3 post-stroke are provided with a prediction for either a Good or Excellent upper limb outcome depending on their age and SAFE score.

Patients with a SAFE score below 5 on day 3 post-stroke are assessed with transcranial magnetic stimulation (TMS) between days 3 and 7 post-stroke. TMS is used to obtain paretic upper limb motor evoked potential (MEP) status, which is a biomarker of functional integrity of the crossed corticospinal tract (CST) from the ipsilesional hemisphere.^4,5^ If MEPs can be elicited in either the paretic extensor carpi radialis (ECR) or first dorsal interosseous (FDI) muscles the patient is considered MEP+ and is predicted to have a Good outcome. If MEPs cannot be elicited from the paretic ECR or FDI then the patient is considered MEP− and receives a prediction for either a Limited or Poor outcome depending on their National Institute of Health Stroke Scale (NIHSS) score obtained on day 3 post-stroke.

PREP2 was developed via Classification and Regression Tree (CART) analysis with 15 potential demographic, clinical, and biomarkers measures. However, several measures that may influence upper limb outcome were not included. These measures include sensory loss in the paretic hand, apraxia, individual SA and FE scores, and whether patients underwent thrombectomy. For example, upper limb apraxia can affect stroke patients’ ability to manipulate and use objects in space^6^ while stroke patients report sensory loss impairs their upper limb function and ability to interact with objects.^7,8^ Several studies have also reported that FE scores alone can predict upper limb motor outcomes.^9,10^ Whether additional measures not included in the original PREP2 development could improve prediction accuracy remains to be determined.

Whether a patient achieves their PREP2 prediction is determined using the Action Research Arm Test (ARAT) at 3 months post-stroke. The ARAT categorizes upper limb function into Excellent, Good, Limited, or Poor outcome categories using cut-off scores determined with a hypothesis-free cluster analysis (Figure 1).^3^ A patient’s PREP2 prediction is considered accurate if their outcome category matches their prediction category.

The PREP2 prediction tool was developed with data from 207 participants after stroke and correctly predicted upper limb outcome for 75% of participants when assessments were performed by researchers.^3^ Accuracy was highest for the Excellent and Poor categories and lowest for the Good category. Most patients’ outcome category at 3 months post-stroke remain the same at 2 years post-stroke.^11^

The PREP2 prediction tool was implemented in routine clinical care at Auckland City Hospital, New Zealand, in 2017 and is now carried out by the patients’ therapy teams.^12^ Implementation involved clinical staff training to perform the SAFE score, NIHSS, and TMS assessment as well as using PREP2 to make and document a prediction, and deliver it to the patient, their family, and the wider clinical team. The use of PREP2 in clinical practice is associated with a shorter length of stay, increases therapist confidence, and helps tailor upper limb therapy content without detrimental effects on functional outcomes.^13^ However, the accuracy of PREP2 predictions made as part of routine clinical care is currently unknown. It is therefore important to assess the accuracy of PREP2 predictions made as part of routine clinical care with clinicians performing the assessments rather than researchers.

This study had 2 aims. The primary aim was to evaluate the accuracy of PREP2 predictions when made by clinicians as part of routine clinical care. This was done by comparing each patient’s PREP2 prediction with their upper limb outcome at 3 months determined using the ARAT. We hypothesized PREP2 would be at least 70% accurate when used in routine clinical care, and if this threshold was met the PREP2 prediction tool would be considered validated in clinical practice. The second aim was to test baseline factors other than those included in the development of PREP2 that may increase prediction accuracy. These factors included sensation, inattention, memory, and communication. These factors were chosen as they could influence upper limb function recovery and outcomes, and they were not included in the original PREP2 analysis. This second aim was exploratory without an a priori hypothesis.

## Methods

### Participants

Patients were recruited between 1st May 2019 and 31st July 2021. The target sample size was 120 participants, with an aim of 30 participants for each of the 4 PREP2 prediction categories. Adults were recruited within 7 days of new ischemic or hemorrhagic stroke in the acute stroke unit at Te Toka Tumai, Auckland City Hospital. Patients were included if they had new upper limb weakness from the index stroke at day 3 post-stroke and were given a PREP2 prediction by the clinical team. Patients were excluded if they were under 18 years old, had a cerebellar stroke, had communication or cognitive deficits precluding informed consent, were medically unwell at the time of recruitment, if they resided out of area precluding follow-up assessments, or had a life expectancy of less than 12 months according to the judgment of their clinical team. Patients with expressive aphasia but without receptive aphasia were eligible for recruitment. All participants provided written informed consent and the study was approved by the regional ethics committee. Data are available from the corresponding author upon reasonable request, and this report was prepared using the Strengthening Reporting of Observational Studies in Epidemiology guidelines (Supplemental Materials).^14^

### PREP2 Prediction Tool

All participants received a PREP2 prediction from their clinical team within the first 10 days post-stroke as part of routine clinical care. The delivery of the prediction involved the patient and their family members being given a general description using standardized language of their expected level of upper limb function at 3M post-stroke, with no reference to ARAT score ranges.^3^ The patient and family members were also given the opportunity to ask questions.^3^ Written information was also provided to participants about their prediction. Researchers were not involved with and did not influence the decisions about which patients should receive a prediction, the assessments for PREP2, or the prediction information provided to patients and their family. Active upper limb therapy dose was recorded for each in-patient rehabilitation session by the treating therapist.

All PREP2 assessments were performed by the clinical team. The SAFE score was obtained on day 1 post-stroke, and days 2 and 3 if needed to make a PREP2 prediction. The NIHSS was obtained on day 3 post-stroke for patients requiring TMS. TMS was performed with a Neuro-MS Monophasic stimulator (Neurosoft, Ivanovo, Russia) between days 3 and 7 post-stroke to obtain MEP status. Surface electromyography was recorded from the paretic ECR and FDI muscles. TMS started at 30% maximum stimulator intensity (MSO) and if no MEPs were elicited then the intensity was increased in increments to 100% MSO. Patients were considered MEP+ if at least 2 MEPs of any amplitude were observed while the participant was at rest or maximally activating both upper limbs, with stimulation up to 100% MSO. If these criteria were not met the patient was considered MEP−. Further details of the TMS protocol for PREP2 can be found in the Supplemental Materials.

### Clinical Assessments

Clinical assessments for the purpose of this study were performed by a trained researcher at baseline within 7 days post-stroke as well as 1, 3, and 6 months post-stroke. Assessments were completed in the hospital for inpatients and either at the University of Auckland or a place of the participant’s choosing for outpatients. The same assessors typically performed the baseline assessments and the follow-up assessments for each participant, and assessors were not blinded to participants’ PREP2 predictions.

The primary outcome measure was the ARAT score at 3M post-stroke. The ARAT was also performed at 1 month (1M) and 6 months (6M) post-stroke. The 3M ARAT score was used to categorize participants’ upper limb outcome using established PREP2 cutoffs.^3^ ARAT scores from 50 to 57 indicate an Excellent outcome, 34 to 49 indicate a Good outcome, 13 to 33 indicate a Limited outcome, and 0 to 12 indicate a Poor outcome. Participants were unaware the ARAT was being used to categorize their upper limb functional outcome, and they were not informed at the 3M or 6M assessments whether they had achieved their PREP2 prediction. Other clinical assessments performed included the Fugl-Meyer Upper Extremity (FM-UE), light touch sensation at the thenar eminence, and the Oxford Cognitive Screen (OCS). Detailed descriptions for all clinical assessments are provided in the Supplemental Materials. Only results relating to the accuracy of PREP2 predictions at 3M are presented here.

### Data Analysis

The OCS evaluates participants’ cognition across 6 domains (language, number, executive thinking, spatial attention, memory, and praxis) which are made up of sub-domains. Established OCS score cut-offs were used to binarize participants as either impaired or intact on each sub-domain.^15^ Participants were considered impaired in any of the 6 domains if they were impaired on any of the corresponding sub-domains. Participants who did not complete an ARAT at 3M post-stroke had their PREP2 outcome category at 3M post-stroke imputed if their 1M and 6M post-stroke outcome categories were identical. Each participant’s total in-patient upper limb therapy time was calculated by summing the active upper limb therapy dose from each rehabilitation session.

### Statistical Analysis

All statistical analyses were performed using IBM Statistical Package for the Social Sciences (Version 29). Overall accuracy for the PREP2 prediction tool was calculated as the number of participants with the same predicted and actual PREP2 outcome categories at 3M post-stroke divided by the total number of participants. The 95% confidence intervals (CIs) for overall accuracy and sub-analyses were determined by categorizing 3M outcomes (better than predicted, accurate predictions, and worse than predicted) and bootstrapping the data for 1000 samples. Positive and negative predictive values (PPV and NPV, respectively) were individually calculated for each PREP2 category.

The second aim of this study was to identify any factors at baseline other than those included in the original development of the PREP2 prediction tool that may increase prediction accuracy. Following the initial statistical analyses, this aim focused on participants with a predicted Good outcome using TMS (TMS Good, n = 22) as prediction accuracy was lowest for this group in the current study. Prediction accuracy was also low for the TMS Good category in the original PREP2 cohort and in a separate cohort where TMS was performed at 2 weeks post-stroke.^3,16^ Due to the small number of TMS Good participants in the current study, 3M outcomes were binarized as Favorable (Excellent or Good outcome) or Unfavorable (Limited or Poor outcome) for these analyses. The 95% CIs for having a favorable outcome were determined by categorizing 3M outcomes (achieved or better than predicted, and worse than predicted) and bootstrapping the data for 1000 samples. The aim was to identify factors that could distinguish between MEP+ patients who did, and did not, achieve a favorable outcome.

A CART analysis was performed. The CART included the novel variables obtained in this study as well as the clinical and demographic variables used to develop the PREP2 prediction tool. The novel variables were sensation (intact, impaired, absent), each of the 6 OCS domains (intact, impaired), thrombectomy (yes, no), and individual SA and FE scores from day 3 post-stroke. These variables were chosen because they were not in clinical use (thrombectomy), were not measured (sensation, 6 OCS domains), or were not included in the CART analysis (SA and FE scores individually) during the original PREP2 development. The variables used to develop the PREP2 prediction tool were sex, age (<80 and ≥80), hemisphere affected (left, right), hand affected (dominant, non-dominant), stroke classification (lacunar infarct, partial anterior circulation infarct, total anterior circulation infarct, posterior circulation infarct, intracerebral hemorrhage), intravenous thrombolysis (yes, no), previous stroke (yes, no), day 3 post-stroke SAFE score, day 3 post-stroke NIHSS score, and total active upper limb therapy dose. The only variables used to develop PREP2 that were not included were MEP status because all TMS Good participants were known to be MEP+, and the MRI biomarkers because they were not collected in the current study. All CART analyses had a maximum tree depth of 3, minimum terminal node size of 4 cases, and “gini” was used to optimize homogeneity within terminal nodes with pruning. One TMS Good participant was excluded from the CART analyses due to incomplete baseline assessments.

The accuracy for TMS Good predictions in the current study was lower than the 53% accuracy for TMS Good predictions observed in the seminal article which introduced PREP2.^3^ In order to evaluate whether participant characteristics could account for this difference in accuracy, the TMS Good participants from the current study were compared with the 36 TMS Good participants from the dataset used to develop the PREP2 prediction tool.^13,17^ The 2 groups were compared for day 3 post-stroke SA, FE, SAFE, and NIHSS scores, in addition to baseline FM-UE and the total amount of active upper limb therapy received. Mann–Whitney *U* tests were used for these comparisons due to non-normality of the data and values of *P* < .05 were considered statistically significant. Values in brackets for accuracies indicate the 95% CIs.

## Results

**Figure 2.**
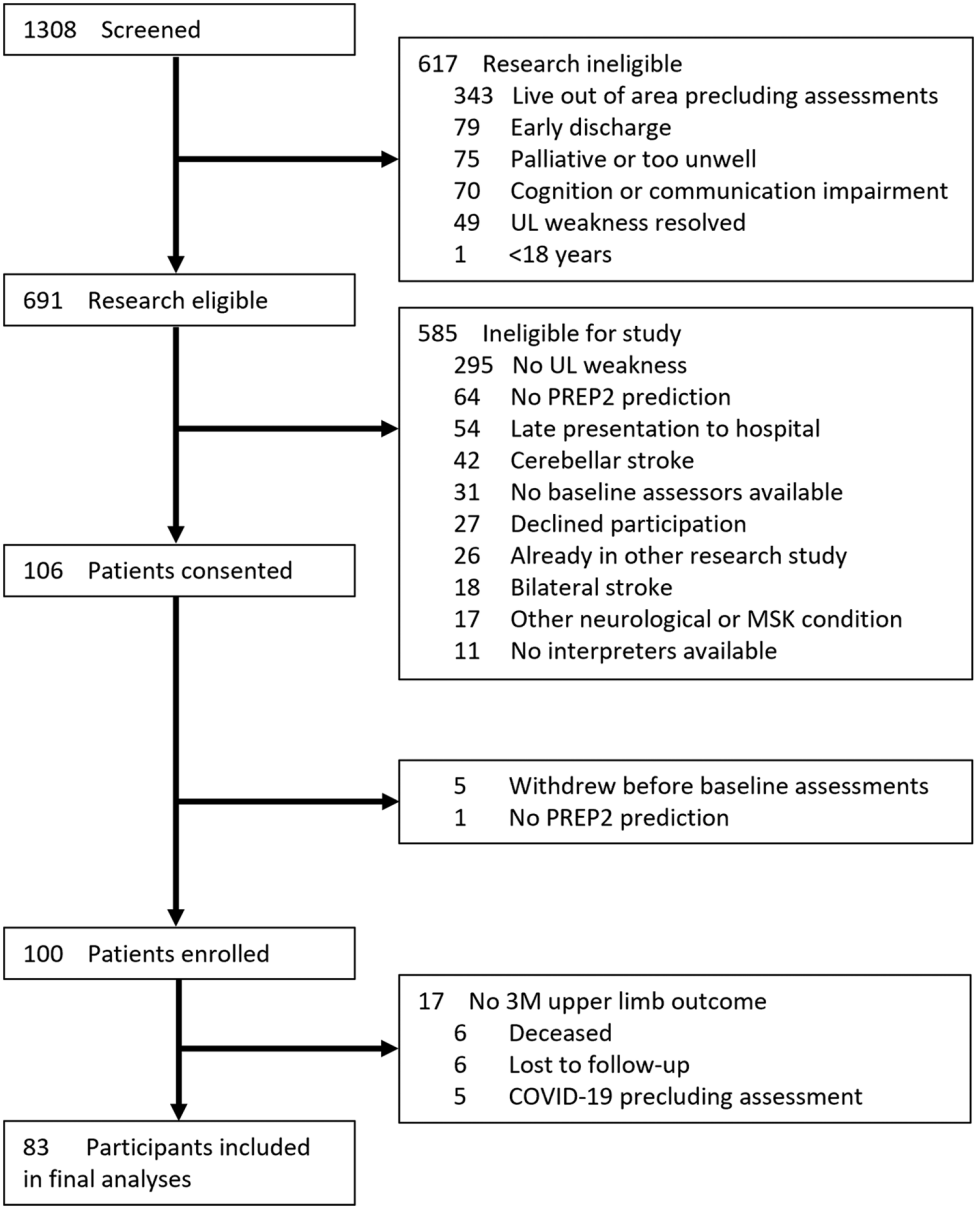
CONSORT diagram of participants.

One hundred participants (56 males [56%]; median age 75 [range: 39-99] years) were enrolled to the study (Figure 2). There were 83 participants with upper limb functional outcome data available at 3M post-stroke who were included in the final analyses (Table 1). Six participants did not complete the 3M post-stroke assessment and had an imputed PREP2 outcome category, 2 of which were Excellent outcomes and 4 of which were Poor outcomes. TMS was required to make a PREP2 prediction for 33 (40%) participants. The median day post-stroke for TMS was 6 days (range: 4-7) and for the NIHSS was 3 days (range: 2-4).

**Table 1.** Demographic and Stroke Characteristics of Participants Included in Statistical Analyses (N = 83).

Demographic characteristics	
Age (years)	
Median age (quartiles, range)	75 (61 – 84, 39 – 99)
Sex	
Male	49 (59%)
Female	34 (41%)
Ethnicity	
Pākehā/NZ European	56 (68%)
Pacific	11 (13%)
Asian	9 (11%)
Māori	7 (8%)
Handedness	
Right	75 (90%)
Pre-admission accommodation	
Home alone	22 (27%)
Home not alone	58 (70%)
Residential care	3 (4%)
Stroke characteristics	
Stroke hemisphere	
Left	45 (54%)
Paretic dominant upper limb	
Yes	48 (58%)
First stroke	
Yes	70 (84%)
Stroke type	
Total anterior circulation infarct	13 (16%)
Partial anterior circulation infarct	18 (22%)
Lacunar infarct	35 (42%)
Posterior circulation infarct	4 (5%)
Intracerebral hemorrhage	13 (16%)
Intravenous thrombolysis	
Yes	8 (10%)
Endovascular thrombectomy	
Yes	6 (7%)
Baseline stroke severity, n = 82	
NIHSS median (quartiles, range)	6 (3 – 9, 0 – 24)
Mild (NIHSS score 0-4)	29 (35%)
Moderate (NIHSS score 5-15)	43 (52%)
Severe (NIHSS score > 15)	10 (12%)
Stroke risk factors	
Hypertension	52 (63%)
Dyslipidemia	16 (19%)
Previous cardiac history	23 (28%)
Atrial fibrillation	21 (25%)
Diabetes mellitus	16 (19%)
Current smoker	12 (15%)
Ex-smoker	16 (19%)
SAFE score day 3 post-stroke	
≥5, does not require TMS	50 (60%)
<5, requires TMS	33 (40%)
PREP2 prediction	
Excellent	44 (53%)
Good	28 (34%)
Limited	2 (2%)
Poor	9 (11%)
Total upper limb therapy dose grouped by PREP2 prediction, minutes, n = 82	
Excellent median (quartiles, range)	30 (10-177, 0-552)
Good median (quartiles, range)	233 (98-423, 0-838)
Limited median (quartiles, range)	366 (357-375, 357-375)
Poor median (quartiles, range)	83 (0-170, 0-335)
Baseline paretic UL measures grouped by PREP2 prediction	
Baseline SAFE score	
Excellent median (quartiles, range)	8 (7-8, 5-10)
Good median (quartiles, range)	2 (0-4, 0-7)
Limited median (quartiles, range)	1.5 (1-2, 1-2)
Poor median (quartiles, range)	0 (0-0.5, 0-1)
Baseline FM-UE score	
Excellent median (quartiles, range)	59 (53-62, 30-65)
Good median (quartiles, range)	14 (10-34, 8-53)
Limited median (quartiles, range)	15 (11-19, 11-19)
Poor median (quartiles, range)	8 (8-10, 8-13)
3M paretic UL measures grouped by PREP2 prediction	
3M FM-UE score, n = 76	
Excellent median (quartiles, range)	63 (60-65, 46-66)
Good median (quartiles, range)	41 (19-57, 10-65)
Limited median (quartiles, range)	27 (13-40, 13-40)
Poor median (quartiles, range)	9 (8-14, 8-24)
3M ARAT score, n = 77	
Excellent median (quartiles, range)	55 (51-57, 37-57)
Good median (quartiles, range)	34 (3-46, 0-57)
Limited median (quartiles, range)	16 (3-29, 3-29)
Poor median (quartiles, range)	0 (0-1, 0-5)

Abbreviations: ARAT, action research arm test; CCI, Charlson Comorbidity Index; FM-UE, Fugl-Meyer Upper Extremity; NIHSS, National Institutes of Health Stroke Scale; PREP2, predict REcovery potential-2; SAFE, shoulder abduction finger extension; TMS, transcranial magnetic stimulation; UL, upper limb.

Stroke types categorized according to the Oxfordshire Community Stroke Project classification system.

### Accuracy of Predictions

The overall accuracy of the PREP2 prediction tool was 66% (95% CI, 55%-76%) as 55 out of 83 predictions were correct (Table 2). There were 4 participants (5%) with an outcome category better than predicted and 24 participants (29%) with an outcome category worse than predicted. The PPV and NPV for each PREP2 category is provided in Table 3. Of the 54 participants who reached or exceeded their prediction at 3M and completed an ARAT at 1M, 39 (72%) already reached their outcome category at 1M post-stroke (Supplemental Table I).

**Table 2.** PREP2 Predictions and 3M Outcome Categories (N = 83).

	3M outcome category			
PREP2 prediction	Excellent	Good	Limited	Poor
Excellent	35	9	0	0
Good	4	10	4	10
Limited	0	0	1	1
Poor	0	0	0	9

Abbreviations: 3M, 3 months post-stroke; PREP2, predict REcovery potential-2.

Each cell is a count of participants.

**Table 3.** PPV and NPV for PREP2 Prediction Categories.

PREP2 prediction	PPV (95% CI)	NPV (95% CI)
Excellent (n = 44)	80% (68-88%)	90% (77-96%)
Good (n = 28)	36% (24-50%)	84% (76-89%)
Limited (n = 2)	50% (7-93%)	95% (93-97%)
Poor (n = 9)	100% (66-100%)	85% (79-89%)

Abbreviations: CI, confidence interval; NPV, negative predictive value; PPV, positive predictive value; PREP2, Predict REcovery Potential-2.

The accuracy for predicted Excellent outcomes was 80% (95% CI, 68%-88%) and the remaining 20% had a Good outcome. Overall accuracy for predicted Good outcomes was 36% (95% CI, 24%-50%), with 67% (95% CI, 25%-100%) accuracy for predictions without TMS and 27% (95% CI, 10%-48%) accuracy for TMS Good predictions. Only 2 participants were predicted to have a Limited outcome and the accuracy was 50% (95% CI, 7%-93%). Accuracy for predicted Poor outcomes was 100% (95% CI, 66%-100%). No MEP− participants had a Good or Excellent outcome. For participants with a SAFE score of 5 or higher on day 3 post-stroke the overall prediction accuracy was 78% (95% CI, 67%-89%, n = 50). For participants with a SAFE score below 5 and who received TMS the overall prediction accuracy was 48% (95% CI, 32%-67%, n = 33). The accuracy of each PREP2 pathway and a Sankey diagram displaying predictions and outcomes are provided in Figures I and II of the Supplemental Materials, respectively.

### Additional Predictive Factors for TMS Good Participants

**Figure 3.**
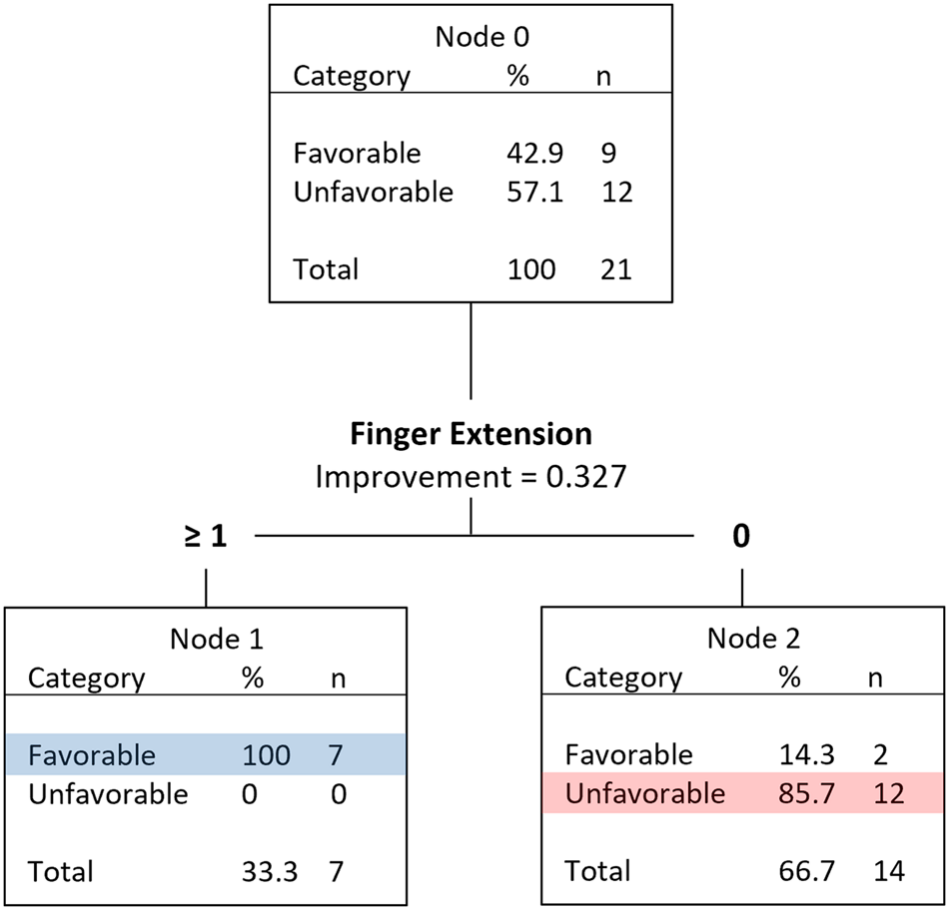
CART analysis for TMS Good participants including novel variables as well as the original clinical and demographic PREP2 variables (n = 21). Abbreviations: CART: Classification and Regression Tree; TMS: transcranial magnetic stimulation; PREP2: Predict REcovery Potential-2.

The CART analysis for the 21 TMS Good predictions produced a decision tree with an overall accuracy of 91% (Figure 3). The only variable identified was FE score. All 7 participants with a FE score >0 had a favorable outcome whereas only 2 of the 14 participants (14%) with a FE score of 0 had a favorable outcome. This indicates that for MEP+ participants having any degree of FE at day 3 post-stroke is an indicator of a favorable upper limb outcome. For MEP+ participants having no FE at day 3 post-stroke is an indicator of an unfavorable upper limb outcome.

### TMS Good Participant Comparison

The accuracy of TMS Good predictions in the current study was 27% (95% CI, 10%-48%) in comparison to the 53% (n = 36, 95% CI, 35%-69%) accuracy for TMS Good predictions in the seminal article which introduced PREP2.^3^ When comparing the TMS Good participants from the current study with the cohort used to originally develop the PREP2 prediction tool, those in the current study had lower SA scores (Mann–Whitney *U* = 243, *P* = .028), lower SAFE scores (Mann–Whitney *U* = 266, *P* = .032), and higher NIHSS scores (Mann–Whitney *U* = 508, *P* = .031). All other comparisons were non-significant (all *P* > .05). More detailed results can be found in Table II of the Supplemental Materials.

## Discussion

The objective of the present study was to assess the accuracy of the PREP2 tool for predicting upper limb functional outcomes at 3 months post-stroke when predictions were made by clinicians as part of routine clinical care. In contrast to our hypothesis, the overall accuracy of PREP2 predictions was 66% (95% CI, 55%-76%). The PREP2 prediction tool is not considered validated in clinical practice as the accuracy of predictions was below the pre-specified 70% accuracy threshold. Prediction accuracy was highest for the Excellent (80% [95% CI, 68%-88%]) and Poor (100% [95% CI, 66%-100%]) categories, and lowest for the Good category (36% [95% CI, 24%-50%]). PREP2 predictions were 78% accurate for the 50 participants with a SAFE score of 5 or higher who did not require TMS. The biggest challenge was participants with a SAFE score less than 5 who required TMS and were MEP+, as only 27% achieved their predicted Good outcome. The exploratory analysis revealed prediction accuracy for these participants could be improved by incorporating binarized measures of finger extension. This study demonstrates that when the PREP2 prediction tool is used as part of routine clinical care it can provide accurate predictions for patients with mild-moderate initial upper limb impairment and for patients with severe initial upper limb impairment who are also MEP−.

This is the first study to evaluate the accuracy of PREP2 when used by clinicians as part of routine care, in contrast to prior studies where PREP2 assessments were performed by researchers attempting to externally validate aspects of the PREP2 prediction tool. Lundquist et al^16^ reported an overall accuracy of 60% when the SAFE score and TMS were performed 2 weeks post-stroke instead of at the correct PREP2 time of 3 to 7 days post-stroke. Barth et al^18^ tested a variation of the PREP2 prediction tool where they obtained SAFE scores at a mean of 1 week post-stroke, did not use TMS, and had a different NIHSS cut-off. The overall prediction accuracy was 61%. Future studies will need to test PREP2 with correct assessment times to externally validate it.

The 66% (95% CI, 55%-76%) accuracy of PREP2 in the current study is lower than the 75% (95% CI, 70%-81%) accuracy observed in the development of PREP2.^3^ Accuracy for the Excellent and Poor categories was similar (80% vs 79% for Excellent, and 100% vs 91% for Poor). The greatest difference in accuracy was for the predicted Good outcome category when TMS was required. For this subgroup, only 41% (95% CI, 21%-64%) of patients achieved a Good or Excellent outcome compared to 78% (95% CI, 63%-90%) of patients in the original study.

There are several potential reasons for the lower accuracy for participants predicted to have a Good outcome using TMS in the current study. One is the level of initial upper limb severity as participants in the current study had greater upper limb impairment compared to the original PREP2 cohort. Another potential reason is that MEP status was determined using a Neurosoft Neuro-MS stimulator in the current study rather than a MagStim 200 stimulator that was used to develop PREP2, with the latter more commonly used in TMS research.^19^ It is possible the Neurosoft Neuro-MS stimulator was able to elicit MEPs in patients with severe CST damage who would have been classified as MEP− using a MagStim 200 stimulator. Stimulator characteristics, including the size of the capacitor, the shape of the coil, and materials used can affect the generated magnetic field and subsequently the ease of eliciting MEPs.^19,20^ These features are inconsistently reported by TMS manufacturers which makes direct comparisons between TMS systems difficult.^19^ Several differences in stimulator characteristics such as duration and rise time of the TMS pulse have been descriptively noted between Neurosoft Neruro-MS and Magstim Bistim stimulators, however no statistical analyses were performed comparing the stimulators.^21,22^

A third explanation is the difference in MEP+ definitions between studies. One of the studies contributing to the original PREP2 cohort categorized patients as MEP+ if MEPs with a peak-to-peak amplitude ≥50 µV could be elicited on at least 4 out of 8 consecutive trials while the muscle remained at rest.^17^ In the current study there was no amplitude requirement, no maximum number of trials delivered, and pre-activation using bilateral facilitation at 100% MSO was performed if MEPs could not be elicited at rest. This means some MEP+ participants in the current study may have been considered MEP− using the previous study’s definition. Future studies or a larger retrospective analysis could be performed to evaluate how the definition of MEP+ influences PREP2 prediction accuracy.

### Predictions Not Requiring TMS

Prediction accuracy was 78% for participants with a day 3 SAFE score of 5 or more. Six of these participants had predicted Good outcomes with an accuracy of 67% (95% CI, 25%-100%). The remaining 44 participants had predicted Excellent outcomes with an accuracy of 80% (95% CI, 68%-88%), similar to the original PREP2 cohort^3^ and higher than cohorts where the SAFE score was obtained at 1 and 2 weeks post-stroke.^16,18^ Although the overall PREP2 prediction tool did not meet the pre-specified threshold of 70% to be considered validated, the upper half of the PREP2 tool not requiring TMS did exceed this threshold.

### MEP+ Participants

Prediction accuracy was only 27% for the 22 participants with a predicted Good outcome using TMS. Therefore, the exploratory aim of the study focused on differentiating between favorable versus unfavorable outcomes for patients with a predicted Good outcome with TMS. The results showed voluntary FE may be a useful discriminator between recovery phenotypes. All 7 participants who scored at least 1/5 for FE had a favorable outcome, while 12 of 14 participants with a score of 0 had an unfavorable outcome.

The current study’s results partially align with previous research investigating voluntary FE as a predictor. Nijland et al^9^ found 89% of participants with flickers of FE but not shoulder abduction had an ARAT score of 10 to 57 at 6 months post-stroke. This high accuracy likely reflects the wide range of outcome scores. Smania et al^10^ reported FE on day 7 post-stroke was related to upper limb outcomes using the 9-hole peg test, FM-UE, and Motricity Index at 3 months post-stroke but their analysis binarized FE between scores of 0 to 3 and 4 to 5 using MRC grades compared to 0 and 1 to 5 in the current study’s analysis. In contrast, Winters et al^23^ showed the absence of FE does not preclude having a favorable upper limb outcome as 45% of participants with no FE at approximately 1 week post-stroke had an ARAT score of at least 10 at 6 months post-stroke, with a median ARAT score of 34. This is higher than the 41% of TMS Good participants who reached a favorable outcome in the current study, but a favorable outcome requires a minimum ARAT score of 34 rather than 10 in Winters et al’s study. Baseline MEP status was not determined by Winters et al so it is unknown how many MEP− participants their sample included. Hoonhorst et al^24^ reported that MEP status had no additional predictive value compared to the SAFE score for predicting whether participants would score at least 22 points on the FM-UE at 6 months post-stroke, but they used stricter criteria than the current study for a MEP+ determination. These prior studies provide evidence in favor of FE as a predictor of binarized upper limb outcomes. The current study indicates that FE may also improve accuracy for Good outcome predictions made with TMS. This needs to be tested in a larger sample before being incorporated in a formal update of the PREP2 prediction tool.

### MEP− Participants

The MEP− status definition in the current study was completely accurate at identifying participants who still had severe upper limb functional impairments at 3 months post-stroke. All 11 MEP− participants in the current study had a Limited or Poor outcome, as predicted by PREP2. In contrast, studies with different MEP status definitions have found some participants labelled MEP− have made significant upper limb recovery.^4,16,24,25^ This discrepancy is likely due to different MEP status definitions as other studies typically record from a single muscle and require a peak-to-peak amplitude criteria of ≧50 μV in either 50% or 100% of trials for a MEP+ determination.^4,24,25^ It is likely that some participants classified as MEP− in these previous studies would be classified as MEP+ using the current study’s definition as it included recording from 2 muscles and active bilateral facilitation from the participant at 100% MSO. However, this more inclusive MEP+ definition in the current study may have come at the cost of a lower accuracy for Good predictions based on MEP+ status. Another contributing factor for the different results between studies may be small sample sizes as several previous studies, as well as the current study, had fewer than 20 MEP− participants.^4,16,24^ These results indicate that the PREP2 TMS protocol and MEP− definition in the current study is extremely accurate at identifying participants who will have severe upper limb functional impairments at 3 months post-stroke.

When examining the PREP2 categories individually, only 2 participants in the current study had a predicted Limited outcome and so accuracy of this category cannot be meaningfully interpreted. The low number of predicted Limited outcomes is consistent with previous studies^3,16^ and indicates that having a SAFE score below 5, being MEP−, and having an NIHSS score lower than 7 is an uncommon combination. Additionally, the current study found only 6% of participants had an ARAT score of 13 to 33 at 3 months post-stroke corresponding to the Limited outcome category and this aligns with previous studies.^16,18,26^ Our understanding of both the predictions and outcomes in the Limited category will be improved by recruiting larger samples and checking the ARAT score boundaries with further hypothesis-free cluster analyses to refine the definition of this small group.

### Clinical Implications

The current study illustrates that the PREP2 prediction tool is feasible to be implemented and used as part of routine clinical care for patients within 1 week of stroke. Given that prediction accuracy was 78% for patients with a SAFE score of 5 or above who did not require TMS, the top half of the PREP2 prediction tool could be implemented clinically in most settings. The most complex assessment in PREP2 is TMS to determine MEP status as TMS is not routinely performed in hospitals. The very high accuracy for MEP− participants demonstrates that the clinical staff, many of whom were new to TMS, were able to successfully learn and perform the PREP2 TMS protocol. If the clinical staff were not sufficiently skilled they may have misclassified some MEP+ patients as MEP−, resulting in some patients being predicted to have a Limited or Poor outcome and having a Good outcome instead. This wasn’t observed, indicating that the clinical staff were able to accurately implement the PREP2 TMS protocol. Most TMS Good participants fell short of their predicted outcome, however from a clinical perspective it is preferable to be too optimistic rather than pessimistic to avoid reducing patient and therapist motivation.

The current study highlights the need to further investigate factors influencing outcomes for patients who require TMS and have a predicted Good outcome. MEP status may be most important to obtain for patients who have a SAFE score below 5 and without any FE on day 3 post-stroke. The high predictive accuracy for MEP− participants indicates that the PREP2 protocol for MEP status determination is able to accurately identify participants who won’t have a favorable upper limb outcome, in contrast to MEP status protocols used in other studies.^16,24,25^ Being able to accurately identify and inform stroke patients who will not make a meaningful upper limb recovery is useful as it helps manage the patient’s expectations, assists the family with discharge planning, and allows therapists to tailor upper limb rehabilitation sessions to minimize disability. Accurately identifying MEP− participants can also help with stratifying participants into upper limb rehabilitation trials, as recommended by international consensus.^27^ The high predictive accuracy for MEP− participants is encouraging but further work is needed to more closely examine patients who are MEP+ using TMS. The influence of the TMS equipment used to determine MEP status could also be evaluated. If prediction accuracy for TMS Good participants depends on the stimulator used it would have implications for hospitals aiming to implement PREP2 into clinical practice.

### Limitations and Future Directions

This study had several limitations. First, the outcome assessors were not blinded to the participant’s PREP2 predictions which introduces the potential for bias. Second, the number of participants predicted to have a Limited outcome was too low to properly assess accuracy for that category. Third, the sample size for the additional analyses was relatively small as it only included 21 participants with a predicted Good outcome using TMS.

The results of this study reveal several future directions. The accuracy of PREP2 predictions made as part of routine care in other clinical environments remains to be determined. Finger extension should be examined along with the PREP2 MEP status definitions in a larger sample of severely impaired patients to see if FE could be incorporated in a future update of the PREP2 tool. Additionally, clinically-feasible TMS measures that are more sophisticated than a binarized MEP status could be explored which may provide higher prediction accuracy for MEP+ patients.^28^ Furthermore, the definition of MEP+ can vary greatly between studies in terms of muscles recorded from, the TMS equipment used, the number of MEPs required, MEP amplitude thresholds, and whether active facilitation is used.^3,24,25,29,30^ Developing a unified definition for MEP status should be prioritized to enable comparisons between upper limb prediction tools as well as their validation.

## Conclusion

This study demonstrates that the PREP2 prediction tool provides accurate predictions for patients with a SAFE score of 5 or higher as well as MEP− patients when it is used as part of routine clinical care. However, this study does not provide validation of the overall PREP2 prediction tool in routine clinical care. Further development of the prediction tool is needed to improve prediction accuracy for patients with a SAFE score below 5 who are MEP+.

## Supplemental Material

sj-docx-1-nnr-10.1177_15459683251412283 – Supplemental material for The Accuracy of the PREP2 Prediction Tool for Upper Limb Outcomes After Stroke as Part of Routine Clinical Care Supplemental material, sj-docx-1-nnr-10.1177_15459683251412283 for The Accuracy of the PREP2 Prediction Tool for Upper Limb Outcomes After Stroke as Part of Routine Clinical Care by Harry Jordan, Olivia Norrie and Cathy M. Stinear in Neurorehabilitation and Neural Repair
