# Supplementary material for: The Accuracy of the PREP2 Prediction Tool for Upper Limb Outcomes After Stroke as Part of Routine Clinical Care
Source: Neurorehabil Neural Repair. 2026 Jan 23;40(3):235–45. doi: 10.1177/15459683251412283 (PMC12936143; doi:10.1177/15459683251412283)
Supplement: sj-docx-1-nnr-10.1177_15459683251412283 – Supplemental material for The Accuracy of the PREP2 Prediction Tool for Upper Limb Outcomes After Stroke as Part of Routine Clinical Care [file sj-docx-1-nnr-10.1177_15459683251412283.docx]

**The Accuracy of the PREP2 Prediction Tool for Upper Limb Outcomes after Stroke as Part of Routine Clinical Care – Supplemental Materials**

**Methods**

**Transcranial magnetic stimulation**

Patients whose day 3 post-stroke SAFE score was below 5 received transcranial magnetic stimulation (TMS) on day 3 – 7 post-stroke to obtain upper limb motor evoked potential (MEP) status for their PREP2 prediction. Surface electromyography (EMG) was recorded from the paretic extensor carpi radialis (ECR) and first dorsal interosseous (FDI) muscles. Standard skin preparation was performed and 45 mm Ag-AgCl electrodes (Cleartrode™ RTL, ConMed, USA) were placed in a belly-tendon arrangement for the ECR while for the FDI they were placed over the muscle belly and dorsum of the paretic hand. A ground strap was placed immediately proximal to the epicondyles of the humerus. The EMG signal was amplified and band-pass filtered (10-1000 Hz), sampled at 2 kHz, and analysed offline using the Neurosoft software (version 4.5, Neurosoft, Ivanovo, Russia).

Monophasic single-pulse TMS was delivered to the ipsilesional primary motor cortex using a figure-of-eight coil (70 mm wing diameter) connected to a Neuro-MS Monophasic stimulator (Neurosoft, Ivanovo, Russia). A posterior-anterior intracranial current was induced by holding the coil on the participant’s scalp at 45° from the midline with the handle pointing posteriorly. Stimulus intensity was initially set to 30% maximum stimulator output (MSO) and increased in increments up to 100% MSO until MEPs were elicited. Different scalp locations around the primary motor cortex of the ipsilesional hemisphere were stimulated at each increasing stimulus intensity to try elicit MEPs. If no MEPs were elicited at 100% MSO with the participant at rest then they were stimulated at 100% MSO while they performed bilateral facilitation by hugging a pillow and attempting to maximally activate their bilateral upper limb muscles. The patient was considered MEP+ by their clinical team if at least 2 MEPs of any amplitude were observed. If this criterion was not met then the patient was considered MEP-. Each MEP status was confirmed by a second clinical TMS assessor. There was no maximum number of stimulations delivered at each intensity, and the patient was deemed MEP- once the TMS operator made the decision that no MEPs could be elicited and they had stimulated all viable scalp locations.

**Clinical assessments**

The Action Research Arm Test (ARAT) is an assessment of upper limb activity limitation that is recommended for stroke trials by international consensus.^1^ The ARAT consists of 19 tasks grouped into grasp, grip, pinch, and gross subscales. Each task is scored on an ordinal scale from 0 – 3.^2^ A score of 3 indicates the task is completed with normal movement. A score of 2 indicates the task is completed but takes abnormally long and/or the patient used compensatory movements. A score of 1 indicates movement is partially performed but the task is not completed. A score of 0 indicates the participant cannot perform any part of the task. The ARAT was scored according to published guidelines ^2^, and normative time limits from older adults without stroke were used to determine whether task completion was slow.^3^ The ARAT was performed at 1-, 3-, and 6-months post-stroke (1M, 3M, and 6M, respectively).

The Fugl-Meyer Upper Extremity (FM-UE) assessment is a valid and reliable measure of unimanual upper limb impairment in people after stroke ^4^. Participants are scored ordinally from 0 – 2 on 33 items with scores of 2 indicating normal performance, 1 indicating partial performance, and 0 indicating the item cannot be performed. Participants completed the FM-UE with their paretic upper limb and were scored according to the protocol by See et al. ^5^. The FM-UE was performed at baseline as well as 1M, 3M, and 6M post-stroke.

The National Institute of Health Stroke Scale (NIHSS) is a measure of stroke severity with 11 items covering motor, sensory, cognitive, communication, and vision symptoms ^6^. The NIHSS is scored out of 42, with higher scores indicating greater stroke severity. The NIHSS was performed on day 3 post-stroke by the participant’s clinical team if it was needed to determine a PREP2 prediction, otherwise it was obtained by research assessors as part of the baseline assessments.

Light touch sensation at the thenar eminence of both hands was assessed using a set of 20 Semmes-Weinstein monofilaments (North Coast Medical, California). A forced-choice three-down one-up staircase protocol with four reversals was used, starting with the 2.83 filament ^7^. Participants were categorised as having impaired sensation if the lightest filament detected by the paretic hand was 2 or more filaments heavier than for the non-paretic hand. Participants were categorised as having absent sensation if they could not detect the heaviest 6.65 filament on their paretic hand. Participants without impaired or absent sensation were categorised as having intact sensation. Light touch sensation was obtained at baseline as well as 3M and 6M post-stroke.

The Oxford Cognitive Screen (OCS) is a cognitive screening tool that assesses six cognitive domains which are language, number, executive thinking, spatial attention, memory, and praxis ^8^. Across the 6 domains there are 12 sub-domains that are evaluated and each has an established cut-off score for impairment. For example, the language domain consists of the “reading,” “semantics,” and “naming” sub-domains and scores below 14/15, 3/3, and 3/4, respectively, indicate impairments on that subdomain. If participants were impaired on a sub-domain they were also considered impaired on the relevant domain. The OCS was performed at baseline as well as 3M and 6M post-stroke.

**Figures and Tables**


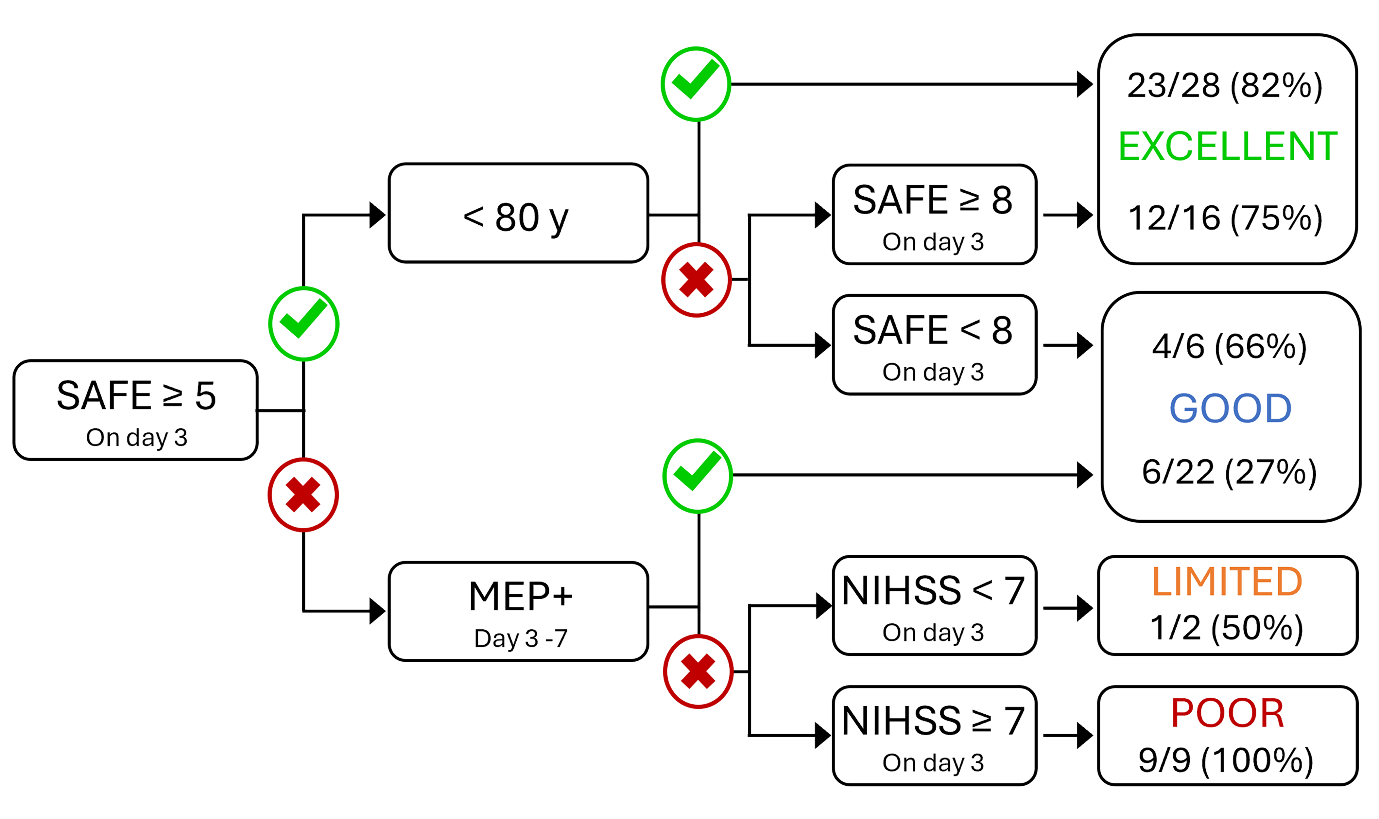


**Figure I.** PREP2 prediction tool accuracy based on individual pathways through the tool (n = 83). The denominator indicates the number of participants who received the indicated PREP2 prediction while the numerator indicates the number of those participants with a correct PREP2 prediction.


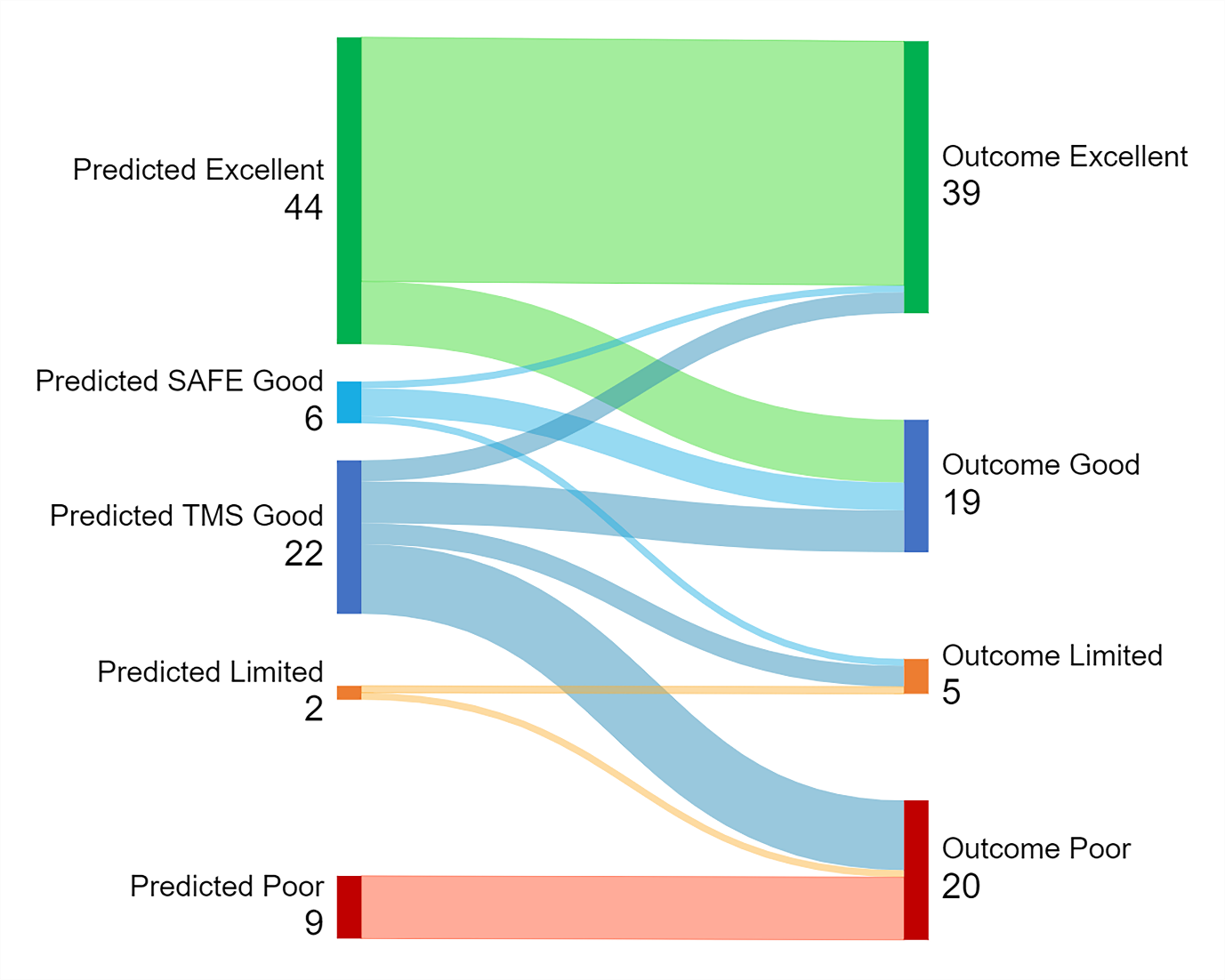


**Figure II.** Sankey diagram of PREP2 predictions and outcomes. The same prediction information was provided to patients with SAFE Good and TMS Good predictions.

**Table I.** 1M and 3M outcome categories for participants who completed an ARAT at 1M and either achieved or exceeded their PREP2 prediction at 3M (n = 54)

|  |  | 3M Outcome Category | | | |
| --- | --- | --- | --- | --- | --- |
|  |  | Excellent | Good | Limited | Poor |
| 1M Outcome Category | Excellent | 26 | 0 | 0 | 0 |
|  | Good | 8 | 4 | 0 | 0 |
|  | Limited | 0 | 4 | 0 | 0 |
|  | Poor | 0 | 2 | 1 | 9 |

Each cell is a count of participants. 1M, 1 month post-stroke; 3M, 3 months post-stroke

**Table II.** Comparison between participants predicted to have a Good outcome using TMS in the current study and participants used to develop the PREP2 prediction tool. p values are from Mann-Whitney U tests. Values are median with range in brackets. * indicates p < 0.05

|  | Current study  (n = 22) | PREP2 development  (n = 36) | p value |
| --- | --- | --- | --- |
| SA ( /5) | 0 (0 – 2) | 2 (0 – 4), n = 33 | 0.028* |
| FE ( /5) | 0 (0 – 2) | 0 (0 – 3), n = 33 | 0.756 |
| SAFE ( /10) | 0.5 (0 – 4) | 2 (0 – 4) | 0.032* |
| NIHSS ( /42) | 9 (3 – 17) | 7 (2 – 16) | 0.031* |
| FM-UE ( /66) | 11.5 (8 – 44) | 21 (2 – 52) | 0.107 |
| Total in-patient UL therapy minutes | 282 (0 – 838) | 333 (32 – 875) | 0.059 |

FE = finger extension, FM-UE = Fugl-Meyer upper extremity, NIHSS = National Institute of Health Stroke Scale, SA = shoulder abduction, UL = upper limb.

**STROBE checklist**

|  | Item Description | Location (or reason for not reporting) |
| --- | --- | --- |
| **Title and abstract** |  |  |
| [1a. Indicate the study’s design](https:/resources.equator-network.org/reporting-guidelines/strobe/items/title-abstract-indicate-study-design.html) | Indicate the study’s design with a commonly used term in the title or the abstract. | Title |
| [1b. Abstract](https:/resources.equator-network.org/reporting-guidelines/strobe/items/abstract.html) | Provide in the abstract an informative and balanced summary of what was done and what was found. | Abstract |
| **Introduction** |  |  |
| [2. Background / rationale](https:/resources.equator-network.org/reporting-guidelines/strobe/items/background-rationale.html) | Explain the scientific background and rationale for the investigation being reported. | Introduction, paragraphs 1-6 |
| [3. Objectives](https:/resources.equator-network.org/reporting-guidelines/strobe/items/objectives.html) | State specific objectives, including any prespecified hypotheses. | Introduction, paragraph 7 |
| **Methods** |  |  |
| [4. Study design](https:/resources.equator-network.org/reporting-guidelines/strobe/items/study-design.html) | Present key elements of study design early in the paper. | Methods |
| [5. Setting](https:/resources.equator-network.org/reporting-guidelines/strobe/items/setting.html) | Describe the setting, locations, and relevant dates, including periods of recruitment, exposure, follow-up, and data collection. | Methods, Participants and Clinical assessments |
| [6a. Eligibility criteria](https:/resources.equator-network.org/reporting-guidelines/strobe/items/eligibility-criteria.html) | **Cohort study:** Give the eligibility criteria, and the sources and methods of selection of participants. Describe methods of follow-up. **Case-control study:** Give the eligibility criteria, and the sources and methods of case ascertainment and control selection. Give the rationale for the choice of cases and controls. **Cross-sectional study:** Give the eligibility criteria, and the sources and methods of selection of participants. | Methods, Participants |
| [6b. Matching criteria](https:/resources.equator-network.org/reporting-guidelines/strobe/items/matching-criteria.html) | **Cohort study:** For matched studies, give matching criteria and number of exposed and unexposed. **Case-control study:** For matched studies, give matching criteria and the number of controls per case. | N/A: This study only included a single group of participants |
| [7. Variables](https:/resources.equator-network.org/reporting-guidelines/strobe/items/variables.html) | Clearly define all outcomes, exposures, predictors, potential confounders, and effect modifiers. Give diagnostic criteria, if applicable. | Methods, PREP2 prediction tool and Clinical assessments |
| [8. Data sources / measurement](https:/resources.equator-network.org/reporting-guidelines/strobe/items/data-sources-measurement.html) | For each variable of interest give sources of data and details of methods of assessment (measurement). Describe comparability of assessment methods if there is more than one group. | Methods, PREP2 prediction tool and Clinical assessments |
| [9. Bias](https:/resources.equator-network.org/reporting-guidelines/strobe/items/bias.html) | Describe any efforts to address potential sources of bias. | Methods, PREP2 prediction tool and Clinical assessments |
| [10. Study size](https:/resources.equator-network.org/reporting-guidelines/strobe/items/study-size.html) | Explain how the study size was arrived at. | Methods, Participants |
| [11. Quantitative variables](https:/resources.equator-network.org/reporting-guidelines/strobe/items/quantitative-variables.html) | Explain how quantitative variables were handled in the analyses. If applicable, describe which groupings were chosen, and why. | Methods, Data analysis |
| [12a. Statistical methods](https:/resources.equator-network.org/reporting-guidelines/strobe/items/statistical-methods-description.html) | Describe all statistical methods, including those used to control for confounding. | Methods, Statistical analysis |
| [12b. Statistical methods – subgroups and interactions](https:/resources.equator-network.org/reporting-guidelines/strobe/items/statistical-methods-subgroups-interactions.html) | Describe any methods used to examine subgroups and interactions. | Methods, Statistical analysis |
| [12c. Statistical methods – missing data](https:/resources.equator-network.org/reporting-guidelines/strobe/items/statistical-methods-missing-data.html) | Explain how missing data were addressed. | Methods, Statistical analysis |
| [12di. Statistical methods – loss to follow-up](https:/resources.equator-network.org/reporting-guidelines/strobe/items/statistical-methods-loss-to-follow-up.html) | **Cohort study:** If applicable, describe how loss to follow-up was addressed. | Methods, Data analysis and Statistical analysis |
| [12dii. Statistical methods – matching cases and controls](https:/resources.equator-network.org/reporting-guidelines/strobe/items/statistical-methods-matching-cases-controls.html) | **Case-control study:** If applicable, explain how matching of cases and controls was addressed. | N/A: This study only included a single group of participants |
| [12diii. Statistical methods – sampling strategy](https:/resources.equator-network.org/reporting-guidelines/strobe/items/statistical-methods-analytical-methods-sampling-strategy.html) | **Cross-sectional study:** If applicable, describe analytical methods taking account of sampling strategy. | N/A: No sampling strategies were used |
| [12e. Statistical methods – sensitivity analyses](https:/resources.equator-network.org/reporting-guidelines/strobe/items/statistical-methods-sensitivity-analyses.html) | Describe any sensitivity analyses. | N/A: No sensitivity analyses were performed |
| **Results** |  |  |
| [13a. Participant numbers](https:/resources.equator-network.org/reporting-guidelines/strobe/items/participants-numbers.html) | Report the numbers of individuals at each stage of the study—e.g., numbers potentially eligible, examined for eligibility, confirmed eligible, included in the study, completing follow-up, and analysed; Consider use of a flow diagram. | Figure 2 |
| [13b. Participants – non-participation](https:/resources.equator-network.org/reporting-guidelines/strobe/items/participants-non-participation.html) | Give reasons for non-participation at each stage. | Figure 2 |
| [13c. Participants – flow diagram](https:/resources.equator-network.org/reporting-guidelines/strobe/items/participants-flow-diagram.html) | Consider use of a flow diagram. | Figure 2 |
| [14a. Descriptive data – participant characteristics](https:/resources.equator-network.org/reporting-guidelines/strobe/items/descriptive-data-participant-characteristics.html) | Give characteristics of study participants (e.g., demographic, clinical, social) and information on exposures and potential confounders. Present the information in a table. | Table 1 |
| [14b. Descriptive data – missing data](https:/resources.equator-network.org/reporting-guidelines/strobe/items/descriptive-data-missing-data.html) | Indicate the number of participants with missing data for each variable of interest. | Table 1 |
| [14c. Descriptive data – follow-up time](https:/resources.equator-network.org/reporting-guidelines/strobe/items/descriptive-data-follow-up-time.html) | **Cohort study:** Summarise follow-up time—e.g., average and total amount. | N/A: This study did not include follow up time. |
| [15. Outcome data](https:/resources.equator-network.org/reporting-guidelines/strobe/items/outcome-data.html) | **Cohort study:** Report numbers of outcome events or summary measures over time. **Case-control study:** Report numbers in each exposure category, or summary measures of exposure. **Cross-sectional study:** Report numbers of outcome events or summary measures. | Results, Accuracy of predictions |
| [16a. Main results](https:/resources.equator-network.org/reporting-guidelines/strobe/items/main-results.html) | Give unadjusted estimates and, if applicable, confounder-adjusted estimates and their precision (e.g., 95% confidence intervals). Make clear which confounders were adjusted for and why they were included. | Results, Accuracy of predictions |
| [16b. Main results – category boundaries](https:/resources.equator-network.org/reporting-guidelines/strobe/items/main-results-category-boundaries.html) | Report category boundaries when continuous variables were categorised. | Methods, Clinical assessments |
| [16c. Main results – risk](https:/resources.equator-network.org/reporting-guidelines/strobe/items/main-results-risk.html) | If relevant, consider translating estimates of relative risk into absolute risk for a meaningful time period. | N/A: Not relevant to this study |
| [17. Other analyses](https:/resources.equator-network.org/reporting-guidelines/strobe/items/other-analyses.html) | Report other analyses done—e.g., analyses of subgroups and interactions, and sensitivity analyses. | Results, Additional predictive factors for TMS Good participants and TMS Good participant comparison |
| **Discussion** |  |  |
| [18. Key results](https:/resources.equator-network.org/reporting-guidelines/strobe/items/key-results.html) | Summarise key results with reference to study objectives. | Discussion |
| [19. Limitations](https:/resources.equator-network.org/reporting-guidelines/strobe/items/limitations.html) | Discuss limitations of the study, taking into account sources of potential bias or imprecision. Discuss both direction and magnitude of any potential bias. | Discussion, Limitations, and future directions |
| [20. Interpretation](https:/resources.equator-network.org/reporting-guidelines/strobe/items/interpretation.html) | Give a cautious overall interpretation considering objectives, limitations, multiplicity of analyses, results from similar studies, and other relevant evidence. | Discussion |
| [21. Generalisability](https:/resources.equator-network.org/reporting-guidelines/strobe/items/generalisability.html) | Discuss the generalisability (external validity) of the study results. | Discussion, Clinical implications |
| **Other information** |  |  |
| [22. Funding](https:/resources.equator-network.org/reporting-guidelines/strobe/items/funding.html) | Give the source of funding and the role of the funders for the present study and, if applicable, for the original study on which the present article is based. | Funding |

**References**

1. Kwakkel G, Lannin NA, Borschmann K, et al. Standardized measurement of sensorimotor recovery in stroke trials: Consensus-based core recommendations from the Stroke Recovery and Rehabilitation Roundtable. *Int J Stroke*. Jul 2017;12(5):451-461. doi:10.1177/1747493017711813

2. Yozbatiran N, Der-Yeghiaian L, Cramer SC. A standardized approach to performing the action research arm test. *Neurorehabil Neural Repair*. Jan-Feb 2008;22(1):78-90. doi:10.1177/1545968307305353

3. Van der Lee JH, De Groot V, Beckerman H, Wagenaar RC, Lankhorst GJ, Bouter LM. The intra- and interrater reliability of the action research arm test: a practical test of upper extremity function in patients with stroke. *Arch Phys Med Rehabil*. Jan 2001;82(1):14-9. doi:10.1053/apmr.2001.18668

4. Sullivan KJ, Tilson JK, Cen SY, et al. Fugl-Meyer assessment of sensorimotor function after stroke: standardized training procedure for clinical practice and clinical trials. *Stroke*. Feb 2011;42(2):427-32. doi:10.1161/STROKEAHA.110.592766

5. See J, Dodakian L, Chou C, et al. A standardized approach to the Fugl-Meyer assessment and its implications for clinical trials. *Neurorehabil Neural Repair*. Oct 2013;27(8):732-41. doi:10.1177/1545968313491000

6. Lyden P, Brott T, Tilley B, et al. Improved reliability of the NIH Stroke Scale using video training. NINDS TPA Stroke Study Group. *Stroke*. Nov 1994;25(11):2220-6. doi:10.1161/01.str.25.11.2220

7. Tracey EH, Greene AJ, Doty RL. Optimizing reliability and sensitivity of Semmes-Weinstein monofilaments for establishing point tactile thresholds. *Physiol Behav*. Feb 28 2012;105(4):982-6. doi:10.1016/j.physbeh.2011.11.002

8. Demeyere N, Riddoch MJ, Slavkova ED, Bickerton WL, Humphreys GW. The Oxford Cognitive Screen (OCS): validation of a stroke-specific short cognitive screening tool. *Psychol Assess*. Sep 2015;27(3):883-94. doi:10.1037/pas0000082
